# The Antiobesity Effect of GLP-1 Receptor Agonists Alone or in Combination with Metformin in Overweight /Obese Women with Polycystic Ovary Syndrome: A Systematic Review and Meta-Analysis

**DOI:** 10.1155/2021/6616693

**Published:** 2021-02-13

**Authors:** Xiaorui Lyu, Taibiao Lyu, Xue Wang, Huijuan Zhu, Hui Pan, Linjie Wang, Hongbo Yang, Fengying Gong

**Affiliations:** ^1^Key Laboratory of Endocrinology of National Health Commission, Department of Endocrinology, Peking Union Medical College Hospital, Chinese Academy of Medical Science, Peking Union Medical College, Beijing 100730, China; ^2^Department of Rheumatology and Clinical Immunology, The Ministry of Education Key Laboratory, National Clinical Research Center for Dermatologic and Immunologic Diseases, Peking Union Medical College Hospital, Chinese Academy of Medical Sciences, Peking Union Medical College, Beijing 100730, China

## Abstract

**Objectives:**

Both glucagon-like peptide-1 receptor agonists (GLP-1RAs) and metformin (MET) have markedly antiobesity effects in overweight/obese polycystic ovary syndrome (PCOS) patients. However, there was no literature to compare the antiobesity effects of these two medicines. Therefore, a systematic review and meta-analysis were conducted in our present study to evaluate the antiobesity effects of GLP-1RAs either as monotherapy or combined with MET in comparison with MET alone in overweight/obese PCOS patients.

**Methods:**

All randomized controlled trials (RCTs) which reported the efficacy of GLP-1RAs and MET in overweight/obese PCOS patients in Medline (from Pubmed), Embase, Cochrane Central Register of Controlled Trials, Web of Science, and Scopus databases were independently searched by two reviewers. The random-effect model was used to pool data extracted from the included literature. The weighted mean difference (WMD) and 95% confidence interval (CI) were used to present the meta-analysis results (PROSPERO registration number: CRD42020173199).

**Results:**

A total of eight eligible RCTs were finally enrolled in our meta-analysis from the 587 retrieved literature. The results showed that GLP-1RAs alone or combined with MET was associated with a greater weight loss (*N* = 318, WMD = −2.61, 95% CI: −3.51 to −1.72, *P* ≤ 0.001, *I*^2^ = 77.5%), more obvious reduction of waist circumference (*N* = 276, WMD = −3.46, 95% CI: −4.36 to −2.56, *P* ≤ 0.001, *I*^2^ = 0.0%), and body mass index (BMI) (*N* = 318, WMD = −0.93, 95% CI: −1.60 to −0.26, *P*=0.007, *I*^2^ = 84.9%) in overweight/obese PCOS patients when compared with MET alone. Further sensitivity analysis demonstrated that the meta-analysis results of the efficacy differences in terms of body weight, waist circumference, and BMI were relatively stable and reliable.

**Conclusion:**

Our meta-analysis demonstrated that the antiobesity effect of GLP-1RAs alone or combined with MET  was superior to MET  alone in terms of weight loss, the reduction of waist circumference, and BMI. More large-scale, high-quality RCTs are needed to further confirm these results in PCOS patients.

## 1. Introduction

Polycystic ovary syndrome (PCOS) is the most common ovarian disorder in women of childbearing age. The incidence rate of PCOS varies from 6.1% to 19.9% among different populations and diagnostic criteria [1, 2]. PCOS is characterized by hyperandrogenism, polycystic ovary, persistent anovulation, and insulin resistance, and more than half of patients are overweight/obese [3, 4]. Overweight/obesity will in turn aggravate insulin resistance and reproductive dysfunction, which further aggravates the occurrence of abnormal glucose and lipid metabolism diseases such as type 2 diabetes (T2DM), hyperlipidemia, and cardiovascular disease in PCOS women [5–9]. Therefore, body weight control of PCOS patients can not only improve the insulin resistance and reproductive function but also reduce the risk of the above diseases [10, 11].

Since the etiology of PCOS is unclear, a healthy diet and physical activity are considered as the mainstay in current status. Besides, owing to the fact that there is a strong association between PCOS and dysglycemia, many hypoglycemic agents are used to manage PCOS women, showing an obvious improvement in terms of impaired glucose tolerance and impaired fasting glucose [12]. Metformin (MET) is the most widely used insulin sensitizer for the treatment of PCOS [13]. After MET treatment, body weight and BMI of PCOS patients decreased significantly with the improvement of insulin resistance, the recovery of regular menstruation, and ovulation function [14–16].

Glucagon-like peptide-1 receptor agonists (GLP-1RAs) are new drugs for T2DM, which can bind to GLP-1 receptors to promote insulin secretion and inhibit glucagon secretion in a glucose-dependent manner, resulting in the restoration of blood glucose control. The benefits of GLP-1RAs are the lower risk of hypoglycemia and cardiovascular disease than other antidiabetic agents [17, 18]. GLP-1RAs also play an important role in the antiobesity treatment [19]. Some prospective RCTs reported the antiobesity effects of GLP-1RAs such as liraglutide (a long-acting GLP-1RA) or exenatide (a short-acting GLP-1RA) in comparison with MET in PCOS patients. Compared with MET, GLP-1RAs can significantly reduce body weight, abdominal obesity and improve the indicators of glucose and lipid metabolism in PCOS patients [20, 21]. However, these studies were single-center RCTs with considerable variation in the quality and results. Moreover, previous meta-analyses focused on the improvement of metabolism and reproductive effect of GLP-1RAs and MET in patients with PCOS [22], and the antiobesity effects of two medicines have not been systematically compared and reviewed, which is also of vital importance for PCOS treatment. In addition, there has been no study comparing the antiobesity effects of GLP-1RAs combined with MET and MET alone in PCOS patients. Therefore, the aim of our study was to evaluate the antiobesity effects of GLP-1RAs either as monotherapy or combined with MET in comparison with MET alone in overweight/obese PCOS patients by using a method of systematic review and meta-analysis.

## 2. Methods

Our study was registered on PROSPERO (PROSPERO registration number: CRD42020173199).

### 2.1. Search Strategy

All randomized controlled trials (RCTs) which compared the efficacy of GLP-1RAs with MET in PCOS patients in Medline (from Pubmed), Embase, Cochrane Central Register of Controlled Trials, Web of Science, and Scopus databases were searched by two reviewers (LXR and LTB) independently from inception until October 2020. The following MeSH terms and relevant terms were used in the search process, including polycystic ovary syndrome, glucagon-like peptide-1 receptor agonist, exenatide, and liraglutide. The language of publication was not limited. The search strategy for Medline (from Pubmed) was presented in the Supplementary Table S1.

### 2.2. Inclusion and Exclusion Criteria

#### 2.2.1. Design

RCTs.

#### 2.2.2. Participants

The patients were overweight/obese premenopausal women diagnosed with PCOS by any recognized diagnostic criteria [3, 23]. The definition of overweight/obesity varied with different national standards. For example, the Chinese standard defined overweight as BMI ≥ 24 kg/m^2^ and obesity as BMI ≥ 28 kg/m^2^ [24].

#### 2.2.3. Intervention and Control

GLP-1RAs (exenatide or liraglutide) alone or combined with MET were compared with MET alone without dosages limitations. The trials that did not use MET as control were excluded.

#### 2.2.4. Outcome

The primary outcome was weight loss. The secondary outcomes were the reduction of waist circumference and BMI. Studies without the primary outcome were also excluded. Any discrepancies between the two reviewers (LXR and LTB) were resolved by consensus with a third reviewer (GFY).

### 2.3. Risk of Bias Assessment

The Revised Cochrane Risk of Bias Tool for Randomized Trials (RoB 2) was used by two reviewers (LXR and LTB) to assess independently the risks of the enrolled studies, including the aspects of the randomization process, the deviations from the intended interventions, the missing outcome data, the measurements of the outcomes, and the selection of the reported results [25].

### 2.4. Data Extraction

Two reviewers established a data extraction form and independently extracted the data from the selected literature. The extracted data focused on general information (author, title, time of publication), participant characteristics (age, country, disease), interventions (GLP-1RAs regimens, dose, duration, and comparison), and predefined outcomes (mean and SD of weight loss, BMI reduction, waist circumference reduction in intervention, and control group). For studies with missing or incomplete outcomes, the corresponding author of these papers was contacted for more information. For data in selected RCTs not presented as we expected, data conversation was performed according to the method recommended in the Cochrane Handbook.

### 2.5. Statistical Analysis

Statistical analysis was performed using Stata 15.1 software. Since all outcomes were continuous variables, weighted mean difference (WMD) and 95% confidence interval (CI) were used to present the results. The *I*^2^ test was used for the heterogeneity test. The random-effect model was used in all analyses regardless of the *I*^2^ value. The metaregression was adopted to explore the sources of heterogeneity. Further sensitivity analysis was conducted to assess whether the results of the meta-analysis were stable or not. *P* < 0.05 was considered to be statistically significant.

## 3. Results

### 3.1. The Study Selection Process and the Final Recruited Studies

**Figure 1:**
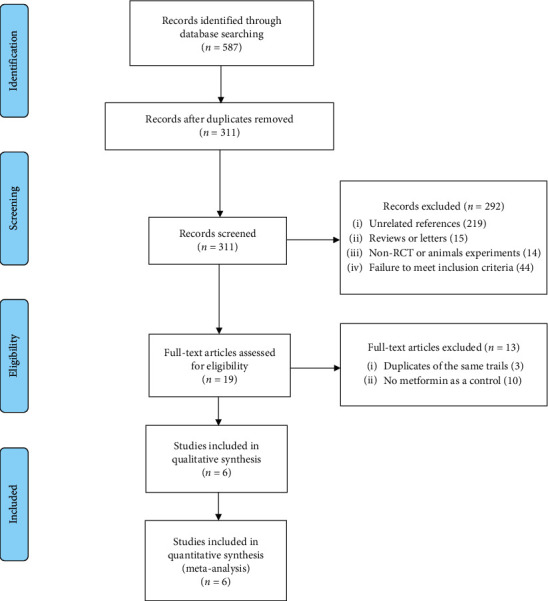
PRISMA flow diagram showing the process of studies selection.

The PRISMA (Preferred Reporting Items for Systematic Reviews and Meta-Analyses) flow chart was used to show the process of the studies selection as presented in Figure 1. After 276 duplicate studies were removed, a total of 311 relevant studies were searched through the databases, of which 292 studies were excluded according to the title and abstract. Then, the remaining nineteen studies were further scrutinized and comprehensively assessed for eligibility. Of these, ten studies were excluded due to the lack of MET treatment as a control, and another three studies were excluded due to the overlapping data with the included studies. Finally, a total of six studies that met the inclusion criteria were enrolled in our meta-analysis, and their detailed information was displayed in references from 25 to 30 [26–31].

### 3.2. The Characteristics of the Enrolled Studies

The characteristics of the enrolled studies were summarized in Tables 1 and 2. Owing to Elkind-Hirsch's [26] and Jensterle Sever's [27], studies had both GLP-1RAs alone treatment and GLP-1RAs in combination with MET treatment in the intervention group; each of the studies was split into two independent trials for analysis. As shown in Tables 1 and 2, eight RCTs reported weight loss and BMI reduction as outcome indicators, while six RCTs provided the waist circumference reduction as an efficacy parameter. The selected RCTs were published between 2008 and 2018. Five RCTs were conducted in Slovenia [27–29, 31], one was in China [30], and two were in America [26]. In addition, five RCTs compared the efficacy differences between GLP-1RAs alone and MET alone, in which a total of 131 patients were treated with GLP-1RAs alone, and 121 patients were treated with MET alone as a control [26–30]. Besides, three RCTs compared the efficacy differences between GLP-1RAs combined with MET and MET alone, in which a total of thirty-eight patients were in GLP-RAs combined with MET treatment and twenty-eight patients were in MET alone treatment [26, 27, 31].

The patients involved in these RCTs were all overweight/obese premenopausal women diagnosed with PCOS according to the revised Rotterdam criteria or National Institute of Child Health and Human Development criteria [3, 23]. As shown in Tables 1 and 2, the category and dosage of GLP-1RAs in these RCTs included liraglutide (1.2 mg once daily (QD)) in the three RCTs [27–29], exenatide (10ug twice daily (BID)) in the two RCTs [26, 30], liraglutide (1.2 mg QD) combined with MET (1000 mg BID) in the two RCTs [27, 31], and exenatide (10 *μ*g BID) combined with MET (1000 mg BID) in one RCTs [26]. MET (1000 mg BID) was selected as a control in all RCTs. In addition, the duration of treatment in the six RCTs was 12 weeks [27–31] and 24 weeks in the other two RCTs [26].

### 3.3. Risk of Bias Assessment

With the RoB2 in Cochrane Handbook, the risks of bias in the recruited studies were assessed from the following five aspects: the randomization process, the deviations from the intended interventions, the missing outcome data, the measurements of the outcomes, and the selection of the reported results. The risk of bias for each study was summarized in Table 3. Among six studies, one study reported the details of the randomization process, one was free of deviations from the intended interventions, four reported the complete outcome data, four were free of selective reporting, and six reported adequate measurements of outcomes. Finally, one study was at low risk [31] and the other five studies had some concerns in the overall risk of bias, as presented in Table 3 [26–30].

### 3.4. A Greater Weight Loss Effect of GLP-1RAs Alone or Combined with MET than MET Alone in Overweight/Obese PCOS Patients

**Figure 2:**
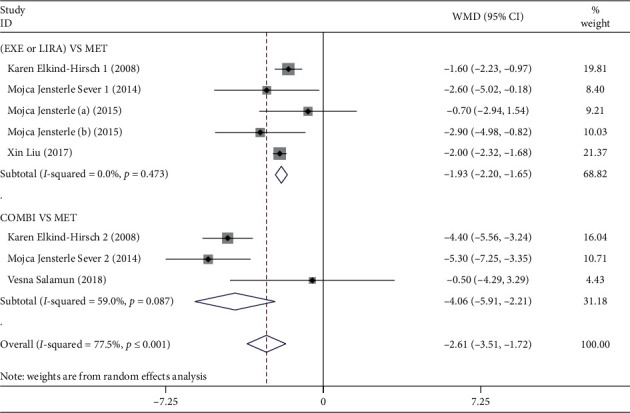
Forest plot of the outcome of weight loss effect in overweight/obese. PCOS patients. EXE: exenatide; LIRA: liraglutide; MET: metformin; COMBI: combination, represented GLP-1RAs combined with MET treatment; WMD: weighted mean difference.

Eight RCTs had reported weight loss of GLP-1RAs alone or combined with MET in comparison with MET alone in overweight/obese PCOS patients as detailed in Tables 1 and 2 [26–31]. As presented in the forest plot of Figure 2, our meta-analysis showed that the weight loss effect of overweight/obese PCOS patients who received GLP-1RAs alone or combined with MET was more significant than patients treated with MET alone (*N* = 318, WMD = −2.61, 95% CI: −3.51 to −1.72, *P* ≤ 0.001, *I*^2^ = 77.5%). Further sensitivity analysis showed that removing anyone of the RCTs had little or no effect on the above result, as presented in Supplementary Figure S1A. In addition, both the Egger's test and Begg's test demonstrated that no publication bias was found in our meta-analysis of weight loss effect (Egger's test: *P*=0.389, Begg's test: *P* ≥ 0.999).

Next, a subgroup analysis was conducted by intervention regimens. The results showed that there was a greater weight loss effect of GLP-1RAs alone than MET alone in overweight/obese PCOS patients (*N* = 252, WMD = −1.93, 95% CI: −2.20 to −1.65, *P* ≤ 0.001, *I*^2^ = 0.0%). Moreover, the effect of GLP-1RAs combined with MET on weight loss was also more obvious than MET alone in overweight/obese PCOS patients (*N* = 66, WMD = −4.06, 95% CI: −5.91 to −2.21, *P* ≤ 0.001, *I*^2^ = 59.0%).

### 3.5. A More Obvious Waist Circumference Reduction Effect of GLP-1RAs Alone or Combined with MET  than MET Alone in Overweight/Obese PCOS Patients

**Figure 3:**
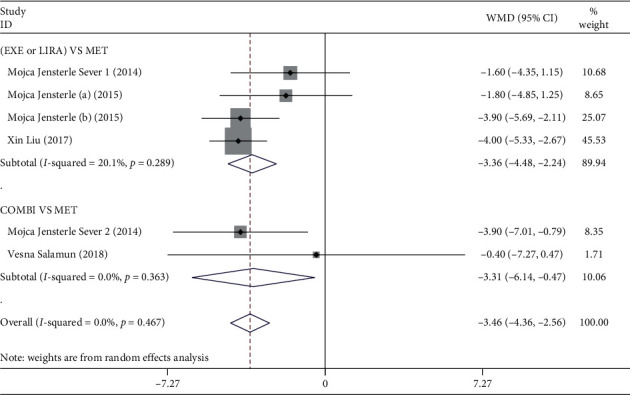
Forest plot of the outcome of waist circumference reduction effect in overweight/obese PCOS patients. EXE: exenatide; LIRA: liraglutide; MET: metformin; COMBI: combination, represented GLP-1RAs combined with MET treatment; WMD: weighted mean difference.

Six RCTs had compared GLP-1RAs alone or combined with MET and MET alone in terms of the waist circumference reduction in overweight/obese PCOS patients as detailed in Tables 1 and 2 [27–31]. The forest plot of the outcome of waist circumference reduction was shown in Figure 3. Our analysis found that GLP-1RAs alone or combined with MET showed a more marked effect on the waist circumference reduction when compared with MET alone in overweight/obese PCOS patients (*N* = 276, WMD = −3.46, 95% CI: −4.36 to −2.56, *P* ≤ 0.001, *I*^2^ = 0.0%). Further sensitivity analysis showed that removing anyone of the RCTs had little or no effect on the above result, as presented in Supplementary Figure S1B. Besides, both Egger's test and Begg's test illustrated that no publication bias was found in our meta-analysis of waist circumference reduction effect (Egger's test: *P*=0.078, Begg's test: *P*=0.452).

Next, the results of subgroup analysis by intervention regimens showed that the waist circumference reduction effect of overweight/obese PCOS patients treated with GLP-1RAs alone was more obvious than those who treated with MET alone (*N* = 231, WMD = −3.36, 95% CI: −4.48 to −2.24, *P* ≤ 0.001, *I*^2^ = 20.1%). As expected, a similar result was observed in the comparison of GLP-1RAs combined with MET and MET alone in overweight/obese PCOS women (*N* = 45, WMD = −3.31, 95%: −6.14 to −0.47, *P*=0.022, *I*^2^ = 0.0%).

### 3.6. A More Significant BMI Reduction Effect of GLP-1RAs Alone or Combined with MET than MET Alone in Overweight/Obese PCOS Patients

**Figure 4:**
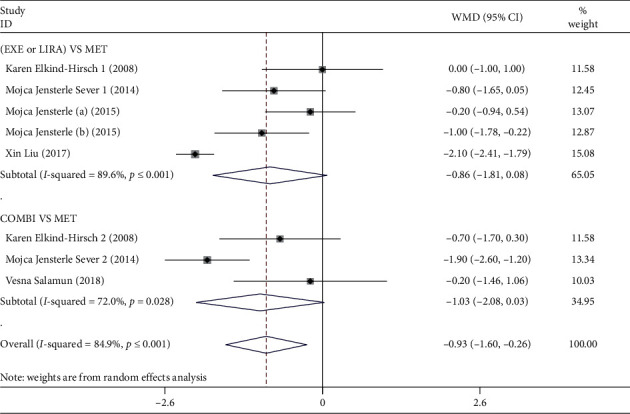
Forest plot of the outcome of BMI reduction effect in overweight/obese. PCOS patients. EXE: exenatide; LIRA: liraglutide; MET: metformin; COMBI: combination, represented GLP-1RAs combined with MET treatment; WMD: weighted mean difference.

Eight RCTs had reported the BMI reduction of GLP-1RAs alone or combined with MET in comparison with MET alone in overweight/obese PCOS patients as detailed in Tables 1 and 2 [26–31]. The forest plot of the outcome of BMI reduction was presented in Figure 4. Our meta-analysis showed that GLP-1RAs alone or combined with MET was associated with a greater BMI reduction effect when compared with MET alone in overweight/obese PCOS patients (*N* = 318, WMD = −0.93, 95% CI: −1.60 to −0.26, *P*=0.007, *I*^2^ = 84.9%). Further sensitivity analysis found that removing anyone of the RCTs had little or no effect on the above result, as detailed in Supplementary Figure S1C. However, the publication bias of the meta-analysis result of BMI reduction was not observed by Begg's test (*P*=0.135), but was observed by Egger's test (*P*=0.004).

In addition, the results of the subgroup analysis by intervention regimens revealed that there was no statistically significant difference between GLP-1RAs alone and MET alone in terms of BMI reduction in overweight/obese PCOS patients (*N* = 252, WMD = −0.86, 95% CI: −1.81 to 0.08, *P*=0.072, *I*^2^ = 89.6%). Similarly, no statistical difference of BMI reduction was also observed between GLP-1RAs combined with MET and MET alone (*N* = 66, WMD = −1.03, 95% CI: −2.08 to 0.03, *P*=0.057, *I*^2^ = 72.0%).

### 3.7. The Sources of Heterogeneity Determined by Metaregression Analysis

The metaregression analysis was conducted to explore the sources of heterogeneity in meta-analysis of weight loss, the reduction of waist circumference, and BMI. The results showed that two different regimens in the intervention group might be one of the sources of heterogeneity in the meta-analysis of weight loss (regression coefficient = −2.68, 95% CI: −4.51 to −0.85, *P*=0.019), suggesting GLP-1RAs alone and GLP-1RAs combined with MET had different degrees of weight loss in comparison with MET alone in overweight/obese PCOS patients. A similar result showed that different population (regression coefficient = −2.35, 95% CI: −3.87 to −0.82, *P*=0.016) might be one of the sources of heterogeneity in the meta-analysis of BMI reduction, suggesting the BMI reduction degree of GLP-1RAs either as monotherapy or combined with MET varied in different populations.

## 4. Discussion

Our meta-analysis evaluated the antiobesity effects of GLP-1RAs either as monotherapy or in combination with MET in comparison with MET alone in overweight/obese PCOS women. The results indicated that GLP-RAs alone or combined with MET were associated with greater weight loss and a more obvious reduction of waist circumference and BMI when compared with MET alone.

As stated in our introduction section, overweight/obesity is a common phenotype in most PCOS patients, and a 5%–10% weight loss can bring obvious benefits in terms of metabolic symptoms and fertility [32]. MET is the first choice to improve insulin resistance for PCOS patients, and it also plays an important role in reducing body weight and BMI. The antiobesity mechanisms of MET are related to various aspects, including the decrease in hepatic gluconeogenesis and insulin synthesis, the inhibition of the hypothalamic appetite regulatory center, and changes in the gut microbiome [33]. GLP-1RAs also play an important role in antiobesity treatment. They can not only inhibit appetite and increase satiety by binding to receptors in the hypothalamus but also delay gastric emptying and inhibit intestinal peristalsis through binding to receptors in gastrointestinal track [34, 35]. Most of our recruiting RCTs showed that both GLP-1RAs and MET could reduce the body weight of PCOS women, with the former demonstrating a greater effect [26–31]. Our meta-analysis also revealed that GLP-1RAs alone or combined with MET showed a greater weight loss effect than MET alone in overweight/obese PCOS patients. Consistently, Lamos' review [36] also illustrated that GLP-1RAs either as monotherapy or combined with MET had a significant effect on weight loss in PCOS women, which summarized eight clinical trials of GLP-1RAs in comparison with placebo or MET in PCOS from PubMed up to 2016. Furthermore, our metaregression analysis found that GLP-1RAs alone and GLP-1RAs combined with MET had different degrees of weight loss in comparison with MET alone in overweight/obese PCOS patients. Elkind-Hirsch's [26] and Jensterle Sever's [27] studies also reported that combination treatment was superior to liraglutide alone or exenatide alone in terms of weight loss in PCOS patients. However, Jensterle's other study [37] showed that high-dose liraglutide had a greater weight loss effect than combination treatment in PCOS women. The inconsistent results might be due to different sample sizes and drug dosage in these studies.

Abdominal obesity is extremely harmful to PCOS patients and hence waist circumference reduction is also an important indicator for evaluating the antiobesity effects of these two medicines [38]. It was reported that both GLP-1RAs and MET had a significant effect in reducing waist circumference in PCOS women in our recruiting RCTs, with the former showing a better effect [26–31]. As expected, our meta-analysis also indicated that GLP-1RAs alone or combined with MET was associated with a more obvious waist circumference reduction effect when compared with MET alone in overweight/obese PCOS patients. Inconsistent with our results, Niafar's meta-analysis showed that waist circumference was not significantly reduced in PCOS women after liraglutide treatment when compared with the control group [39]. The following reasons might explain the inconsistent results: Firstly, the GLP-RAs regimens in the intervention group were different. Niafar's study included liraglutide treatment only, while exenatide treatment, liraglutide combined with MET treatment, and exenatide combined with MET treatment were also included in our study. Secondly, the study types recruited in the meta-analysis were different. Niafar's study considered a case-control study [40], while all selected studies in our analysis were RCTs.

BMI reduction is also considered a secondary outcome for a comprehensive assessment of the antiobesity effects of these two medicines in overweight/obese PCOS patients. Most of our selected RCTs indicated that both GLP-1RAs and MET had a significant effect of reducing BMI in PCOS patients, with the former having a greater effect [26–31]. Our meta-analysis also demonstrated that the BMI reduction effect in overweight/obese PCOS patients who received GLP-1RAs alone or combined with MET was superior to those who received MET alone. Consistent with our results, Niafar's meta-analysis found that BMI of PCOS patients had significantly dropped after liraglutide treatment in comparison with the control group [39]. Besides, Han's meta-analysis also showed that GLP-1RAs was more effective in reducing BMI in PCOS women when compared with MET [22]. However, our result of the subgroup analysis by intervention regimens indicated that there was no statistically significant difference between any subgroups and the control group in overweight/obese PCOS patients. This might be related to the smaller and larger variation of sample size in the subgroups after grouping. In addition, our metaregression analysis showed GLP-1RAs, either as monotherapy or in combination with MET, had different degrees of BMI reduction in various populations, suggesting that PCOS patients' race should be taken into consideration in the clinical use of GLP-1RAs.

In terms of safety, differences in terms of adverse event incidence between GLP-1RAs or combined with MET and MET alone were not evaluated in our study. However, Lamos' review [36] showed GLP-1RAs were well-tolerated, and the most significant adverse side effect was nausea. Han's meta-analysis [22] reported that the incidence of nausea and headache was higher in PCOS patients treated with GLP-RAs than those who were treated with MET, and there was no difference in other side effects.

To the best of our knowledge, this was the first systematic review and meta-analysis which comprehensively evaluates the antiobesity effects of two regimens—GLP-1RAs alone or combined with MET and MET alone in overweight/obese PCOS patients. Our meta-analysis showed that GLP-1RAs combined with MET was a better choice for PCOS patients who had a poor response to GLP-1RAs alone or MET alone in terms of weight loss. Moreover, PCOS patients' race should be taken into consideration in the clinical use of GLP-1RAs in order to have a better effect in terms of BMI reduction. However, this study also has some limitations. First of all, the selected RCTs in the meta-analysis of weight loss degree and BMI reduction degree had considerable heterogeneity. Despite the significant heterogeneity of those studies, sensitivity analysis showed that the results of the above meta-analysis were relatively stable and reliable. Secondly, Egger's test showed that there might be a potential publication bias in the meta-analysis of BMI reduction degree. However, the number of RCTs recruited in our study was small, so the significance of publication bias was limited. In addition, there were different definitions of overweight/obesity in the included studies, which might have an impact on our results.

## 5. Conclusion

Taken together, our meta-analysis provided evidence that the antiobesity effects of GLP-1RAs alone or combined with MET were superior to MET alone in terms of weight loss, reduction of waist circumference, and BMI. However, the sample size is selected RCTs was small, and most of the RCTs were of moderate quality. More large-sample, high-quality RCTs are needed to further confirm these results in overweight/obese PCOS patients in the future.

## Figures and Tables

**Table 1:** Characteristics of the studies included in the meta-analysis.

Study ID	Trial identifier	Author	Year	Country	Disease	Duration (W)
1	Not available	Elkind-Hirsch-1	2008	USA	PCOS	24
2	Not available	Elkind-Hirsch-2	2008	USA	PCOS	24
3	NCT01911468	Jensterle Sever-1	2014	Slovenia	PCOS	12
4	NCT01911468	Jensterle Sever-2	2014	Slovenia	PCOS	12
5	NCT01899430	Jensterle (a)	2015	Slovenia	PCOS	12
6	NCT02187250	Jensterle (b)	2015	Slovenia	PCOS	12
7	ChiCTR-IIR-16008084	Liu xin	2017	China	PCOS	12
8	NCT03353948	Salamun	2018	Slovenia	PCOS	12

**Table 2:** Characteristics of the studies included in the meta-analysis.

Study ID	Intervention						Control					
	Type (dose)	N	Age	BWB (kg)	WCB (cm)	BMIB (kg/m^2^)	Type (dose)	N	Age	BWB (kg)	WCB (cm)	BMIB (kg/m^2^)
				(BWC)	(WCC)	(BMIC)				(BWC)	(WCC)	(BMIC)
1	EXE (10 *μ*g BID)	14	28.2 ± 4.9	110.5 ± 26.8 (−3.2 ± 0.4)		40.3 ± 8.9 (−1.0 ± 1.1)	MET (1000 mg BID)	7	27.7 ± 5.8	113.4 ± 31.3 (−1.6 ± 0.8)		43.3 ± 8.9 (−1.0 ± 1.1)
2	EXE (10 *μ*g BID) + MET (1000 mg BID)	14	32.1 ± 3.1	112.0 ± 35.8 (−6.0 ± 1.9)		40.9 ± 8.9 (−1.7 ± 1.1)	MET (1000 mg BID)	7	27.7 ± 5.8	113.4 ± 31.3 (−1.6 ± 0.8)		43.3 ± 8.9 (−1.0 ± 1.1)
3	LIRA (1.2 mg QD)	11	31.5 ± 6.4	108.9 ± 15.1 (−3.8 ± 3.7)	124.9 ± 9.9 (−3.2 ± 2.9)	39.3 ± 4.2 (−1.3 ± 1.3)	MET (1000 mg BID)	7	31.3 ± 9.4	103.2 ± 6.3 (−1.2 ± 1.4)	122.3 ± 7.0 (−1.6 ± 2.9)	36.6 ± 3.5 (−0.5 ± 0.5)
4	LIRA (1.2 mg QD) + MET (1000 mg BID)	11	31.1 ± 5.1	105.5 ± 20.6 (−6.5 ± 2.8)	121.9 ± 17.7 (−5.5 ± 3.8)	37.6 ± 5.1 (−2.4 ± 1.0)	MET (1000 mg BID)	7	31.3 ± 9.4	103.2 ± 6.3 (−1.2 ± 1.4)	122.3 ± 7.0 (−1.6 ± 2.9)	36.6 ± 3.5 (−0.5 ± 0.5)
5	LIRA (1.2 mg QD)	14	29.5 ± 7.7	113.7 ± 18.7 (−3.0 ± 3.4)	128.5 ± 13.9 (−4.4 ± 5.0)	41.6 ± 5.3 (−1.1 ± 1.1)	MET (1000 mg BID)	14	25.3 ± 5.2	103.6 ± 19.7 (−2.3 ± 2.6)	121.6 ± 17.1 (−2.6 ± 3.0)	37.4 ± 6.4 (−0.9 ± 0.9)
6	LIRA (1.2 mg QD)	14	30.7 ± 7.9	102.8 ± 16.3 (−3.1 ± 3.5)	115.7 ± 12.5 (−3.1 ± 2.8)	36.7 ± 5.6 (−1.1 ± 1.3)	MET (1000 mg BID)	13	30.7 ± 7.9	108.3 ± 17.0 (−0.2 ± 1.8)	120.5 ± 14.5 (0.8 ± 1.9)	39.4 ± 6.9 (−0.1 ± 0.7)
7	EXE (10 *μ*g BID)	80	27.9 ± 2.7	73.0 ± 9.8 (−4.3 ± 1.3)	93.0 ± 10.1 (−9.0 ± 3.8)	29.2 ± 3.1 (−3.1 ± 1.4)	MET (1000 mg BID)	78	27.7 ± 3.8	70.4 ± 4.6 (−2.3 ± 0.6)	89.4 ± 6.6 (−5.0 ± 4.7)	28.3 ± 1.9 (−1.0 ± 0.2)
8	LIRA (1.2 mg QD) + MET (1000 mg BID)	14	30.1 ± 3.6	106.6 ± 11.7 (−7.5 ± 3.9)	114.5 ± 9.9 (−11.7 ± 9.0)	37.8 ± 3.0 (−2.7 ± 1.3)	MET (1000 mg BID)	14	31.1 ± 4.7	99.6 ± 17.8 (−7.0 ± 6.0)	108.8 ± 14.5 (−11.3 ± 9.2)	35.5 ± 4.9 (−2.5 ± 2.0)

BID: twice daily; QD: once daily; BWB: body weight baseline (kg); BWC: body weight changes (kg); WCB: waist circumference baseline (cm); WCC: waist circumference changes (cm); BMIB: body mass index baseline (kg/m^2^); BMIC: body mass index changes (kg/m^2^); EXE: exenatide; LIRA: liraglutide; MET: metformin.

**Table 3:** Summary of the risk of bias for each study according to Revised Cochrane Risk of Bias Tool for Randomized Trials (RoB 2).

Study	Risk of bias due to					Overall risk of bias
	The randomization process	Deviations from the intended interventions	Missing outcome data	Measurements of the outcomes	Selection of the reported results	
Elkind-Hirsch et al. [26]	Some concerns	Some concerns	Low risk	Low risk	Low risk	Some concerns
Jensterle Sever et al. [27]	Some concerns	Some concerns	Low risk	Low risk	Low risk	Some concerns
Jensterle et al. [28]	Some concerns	Some concerns	Low risk	Low risk	Low risk	Some concerns
Jensterle et al. [29]	Some concerns	Some concerns	Some concerns	Low risk	Some concerns	Some concerns
Liu et al. [30]	Some concerns	Some concerns	Some concerns	Low risk	Some concerns	Some concerns
Salamun et al. [31]	Low risk	Low risk	Low risk	Low risk	Low risk	Low risk

## Data Availability

The data used in our study are available from the corresponding author upon request.

## Supplementary Materials

Supplementary Materials Supplementary Table S1: the overall search strategy for Medline (from PubMed) in our meta-analysis. Supplementary Figure S1: sensitivity analysis of meta-analysis outcomes for weight loss effect (A), waist circumference reduction effect (B), and BMI reduction effect (C).
