# Supplementary material for: The Antiobesity Effect of GLP-1 Receptor Agonists Alone or in Combination with Metformin in Overweight /Obese Women with Polycystic Ovary Syndrome: A Systematic Review and Meta-Analysis
Source: Int J Endocrinol. 2021 Feb 13;2021:6616693. doi: 10.1155/2021/6616693 (PMC7910049; doi:10.1155/2021/6616693)

**Supplementary material s**

**Table.S1 The overall search strategy for Medline (from Pubmed) in our meta-analysis**

| **Step** | **Search strategy** |
| --- | --- |
| #1 | (((Glucagon-Like Peptide-1 receptor agonist[Title/Abstract]) OR GLP-1 receptor agonist[Title/Abstract]) OR GLP-1 RA[Title/Abstract]) OR Glucagon-Like Peptide-1 receptor agonist[MeSH Terms] |
| #2 | (((((exenatide[MeSH Terms]) OR exenatide[Title/Abstract]) OR Bydureon[Title/Abstract]) OR Exendin-4[Title/Abstract]) OR EXE[Title/Abstract]) OR AC 2993[Title/Abstract] |
| #3 | ((((((liraglutide[Title/Abstract]) OR liraglutide[MeSH Terms]) OR LIRA[Title/Abstract]) OR Saxenda[Title/Abstract]) OR NN-2211[Title/Abstract]) OR liraglutide recombinant[Title/Abstract]) OR Victoza[Title/Abstract] |
| #4 | #1 OR #2 OR #3 |
| #5 | ((((Polycystic ovary syndrome[Title/Abstract]) OR Polycystic ovary syndrome[MeSH Terms]) OR Stein-Leventhal Syndrome[Title/Abstract]) OR Sclerocystic Ovaries[Title/Abstract]) OR PCOS[Title/Abstract] |
| #6 | #4 AND #5 |

**Fig.S1 Sensitivity analysis of meta-analysis outcomes for weight loss effect (A), waist circumference reduction effect (B) and BMI reduction effect (C).**


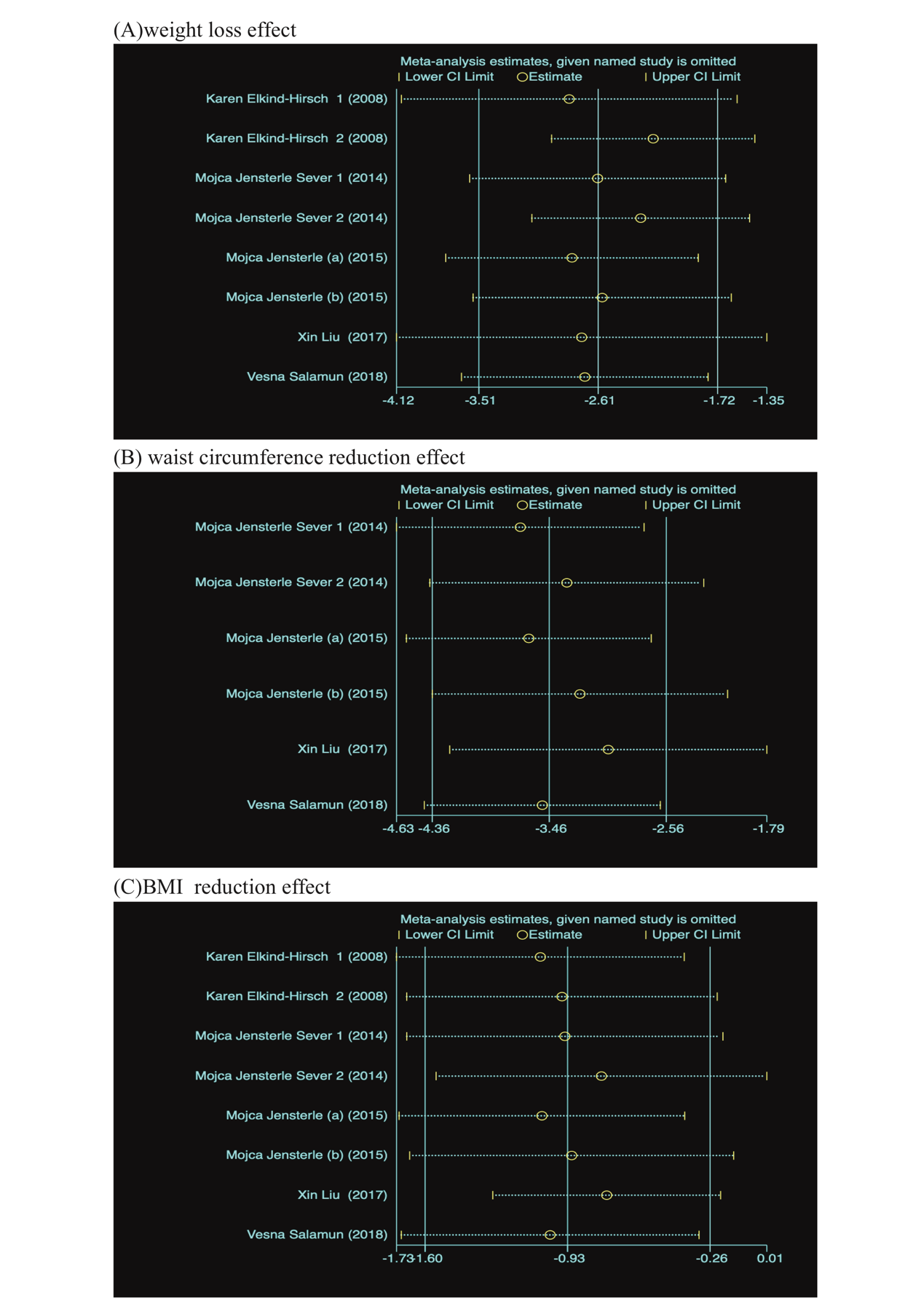

Supplement: Supplementary Materials — Supplementary Table S1: the overall search strategy for Medline (from PubMed) in our meta-analysis. Supplementary Figure S1: sensitivity analysis of meta-analysis outcomes for weight loss effect (A), waist circumference reduction effect (B), and BMI reduction effect (C). [file 6616693.f1.docx]
